# Bilirubin Protects Transplanted Islets by Targeting Ferroptosis

**DOI:** 10.3389/fphar.2020.00907

**Published:** 2020-06-16

**Authors:** Qing Yao, Rui Sun, Shihui Bao, Ruijie Chen, Longfa Kou

**Affiliations:** ^1^Department of Pharmacy, The Second Affiliated Hospital and Yuying Children’s Hospital of Wenzhou Medical University, Wenzhou, China; ^2^School of Pharmaceutical Sciences, Wenzhou Medical University, Wenzhou, China

**Keywords:** bilirubin, ferroptosis, islet transplantation, oxidative stress, HO-1/Nrf2

## Abstract

Islet transplantation is an attractive treatment for type 1 diabetic patients. However, transplanted islets suffered from considerable cell death due to inflammatory reactions and oxidative stress. Ferroptosis is a programmed death characterized by iron-dependent lipid peroxidation, which has been implicated in the islet loss and dysfunction. Our previous studies showed that bilirubin displayed protection effect for islets by inhibiting early inflammation and oxidative stress. However, whether bilirubin protects islets by targeting ferroptosis has not yet been elucidated. Here, the isolated islet was exposed to ferroptosis-inducing agents with or without bilirubin. Islet viability, insulin secretion, and oxidative stress levels were assessed. Subsequently, the pretreated islets were transplanted into the subrenal site of streptozotocin-induced diabetic mice. Bilirubin could significantly attenuate ferroptosis in isolated islets, along with reduced oxidative stress, elevated GPX4 expression and upregulation of Nrf2/HO-1. Experimental data also confirmed that bilirubin could chelate iron. In vivo graft study demonstrated that euglycemia was achieved in diabetic mice receiving bilirubin-pretreated islets within 24 hours, while the control islets required at least 7 days. Bilirubin could improve islet viability and function through inhibiting ferroptosis, which could be of clinic interest to apply bilirubin into the islet transplantation system.

## Introduction

Transplantation of human pancreatic islets is a viable method for patients with complicated type 1 diabetes mellitus (TIDM) (Farney et al., 2016). However, transplanted islets suffered from considerable cell death due to hypoxia-induced inflammatory reactions and oxidative stress, which limited the application of islet transplantation. Therefore, medical approaches to increase the number of viable and functional transplanted islets are of clinic interest for late state T1DM patients (Shapiro et al., 2017). Islets are genetically more susceptible to hypoxia-induced oxidative stress due to the poor antioxidative defense system (Gerber and Rutter, 2017). Additionally, ischemia injury is an evitable event after cell transplantation, which leads to a shortage of oxygen and nutrients for islet survival.

Ferroptosis is a recently discovered pathway of regulated cell death, which characterized by the iron-dependent increase in oxidative stress and lipid peroxidation (Xie et al., 2016). This genetically determined and programmed process is believed to cause the impaired function of islets and promote the necrotic debris immunogenicity (Bruni et al., 2018a). Bruni et al. have demonstrated that ferroptosis plays an important role in pancreatic islet loss during isolation, culture, and transplantation, and ultimately affect the graft efficacy (Bruni et al., 2018b). Researchers have shown that ferroptosis can be inhibited by the ferroptosis specific inhibitors, e.g., ferrostatin-1 (Ferrin-1), ferroptosis regulator glutathione peroxidase 4 (GPX4), and iron chelating agents, e.g., deferoxamine (DFO) (Dixon, 2017). It is viable option to protect the isolated islets by targeting ferroptosis and inhibiting the formation of iron-dependent lipid peroxidation (Bruni et al., 2018b). Interestingly, the DFO treatment has been reported to somehow protect transplanted islets in the 1980s (Bradley et al., 1986), but the mechanism was not clear until the concept of ferroptosis came up. Thus, the involvement of ferroptosis helps to validate the application of DFO in the islet transplantation, which inspires us to wonder if other therapeutics that have been reported to protect transplanted islets also take action by targeting ferroptosis.

Bilirubin, an endogenous heme metabolite, could exert protection effect for islets against non-specific inflammation and oxidative stress (Kim et al., 2017a; Bae et al., 2018; Pennell et al., 2019). Pretreatment with bilirubin improved the islet resistance against excessive reactive oxygen species (ROS), or proinflammatory cytokines mediated cell death (He et al., 2015; Fullagar et al., 2017; Yao et al., 2019a). In vivo results showed that bilirubin treatment remarkably improved allografted islet survival and decreased graft rejection in diabetic mice (Fullagar et al., 2017; Yao et al., 2019b). Researchers indicated that the protective role of bilirubin for isolated islets was associated with its sufficient ROS scavenging ability (Kim et al., 2017a; Bae et al., 2018; Pennell et al., 2019). Our previous study also showed that bilirubin displayed protection effect for the islets against oxidative stress by reducing the ROS levels and modulating the levels of intracellular antioxidative enzymes, including glutathione peroxidase (GPX) and superoxide dismutase (SOD) (Yao et al., 2019b). Additionally, bilirubin could chelate many metal ions, including iron, forming a metal complex, which has been widely used for biosensor and theranostic (Nandi and Biswas, 2019; Shanmugaraj and John, 2019; Srinivasan et al., 2019; Xing et al., 2019). As introduced, ferroptosis is a form of regulated cell death that is dependent on iron and ROS (Xie et al., 2016). Thus, we have reasons to hypothesize that bilirubin plays a protective role by modulating the ferroptosis process in transplanted islets. However, there is still no definitive evidence that bilirubin could target ferroptosis and exert a cytoprotective effect.

The aims of current study were (i) to study the effect of ferroptosis on islet survival and function; (ii) to assess the protective effects and involved mechanisms of bilirubin in ferroptosis induced islet loss and dysfunction; (iii) to evaluate and compare the in vivo performance of bilirubin or ferroptosis inhibitors protected islets transplanted to the diabetic mouse. In this study, isolated mouse islets were exposed to ferroptosis inducing agents with or without bilirubin pretreatment, and their cell survival, insulin secretion, intracellular free radical, lipid peroxidation, and in vivo islet graft outcomes were evaluated. Additionally, the iron-chelating property of bilirubin was also studied to provide additional information.

## Materials and Methods

### Islet Isolation and Cultivation

Institutional Animal Care and Use Committee approved all animal experiments. Male BALB/c mice, 6 to 8 weeks old, weighing at 20 to 25 g, were purchased from the Animal Research Center at Wenzhou Medical University. Mice were anesthetized before laparotomy. A collagenase (Type IV) (Sigma-Aldrich, St.Louis, MO, USA) digestion method which has been reported in our previous studies were utilized for islet isolation and cultivation (Yao et al., 2019a; Yao et al., 2020a). In brief, before sacrifice, the mouse pancreas was distended in collagenase IV contained Hanks' balanced salt solution (HBSS). Following that, collagenase IV was used to digest the isolated pancreatic tissue in a 50 mL tube maintained at 37°C for 5 min. Afterwards, we used a density gradient medium to purify the pancreatic islets and further collected them by handpicking using an optical microscope. In addition, Min6 cells were purchased from the cell bank of type culture collection of Chinese Academy of Sciences (Shanghai, China) for some experiments, and the cells have been authenticated and tested for mycoplasma contamination.

### Drug Treatment

This Erastin (purity≥98%, Sigma-Aldrich, Shanghai, CN) and Ferrin-1 (purity≥95%, Sigma-Aldrich, Shanghai, CN) stock solution at a concentration of 10 mM used for culturing islets or Min6 cells were prepared in PBS. DFO (purity≥92.5%, Sigma-Aldrich, Shanghai, CN) stock solution at a concentration of 75 mM used for experiments were prepared by dissolving in sodium hydroxide solution. Bilirubin (purity≥98%, Sigma-Aldrich, Shanghai, CN) was firstly dissolved in sodium hydroxide, following with adjusting to neutral pH (7.4) using hydrochloric acid, and then the solution was diluted to 1 mM as a final concentration. The stock solutions were all stored at -4°C.

### Islet Survival

Fluorescein diacetate (FDA) and propidium iodide (PI) were used to evaluate the islet survival as previously reported (Yao et al., 2019a). The FDA and PI staining (FDA, 10 µg/mL; PI, 0.5 mg/mL) were observed under fluorescence microscopy (DMi8-M, Leica). The fluorescent images were recorded, and the quantitative analysis was performed by using Image J software. The survival rate of islets was calculated as the following equation.

$$The\;islet\;survival\;rate\;\left(\%\right)=\frac{A_{FDA}}{A_{FDA}+A_{PI}}\times 100\%$$

*A_FDA_* was the FDA stained area, and *A_PI_* was PI stained area.

### Glucose-Stimulated Insulin Secretion (GSIS) Assay

GSIS was performed as previously reported to assess the islet function (Yao et al., 2019a). After drug treatment, islets were collected for GSIS assay. Briefly, 150 islet equivalents (IEQs) were incubated in HEPES buffer with BSA containing 2.8 mM (low) or 28 mM (high) glucose for 1 hour at 37 °C, respectively. Then, the cells were harvested, with the insulin concentration determined by an enzyme-linked immunosorbent assay (ELISA) kit (Alpco Diagnostics, Salem, USA). The stimulation index (SI) was calculated as the following equation.

$$The\;stimulation\;index\;\left(SI\right)=\frac{C_{high\;glucose}}{C_{low\;glucose}}$$

C_high-glucose_ was the secreted insulin concentration of islets under high-glucose stimulation , and C_low-glucose_ was the secreted insulin concentration of islets under low-glucose stimulation.

### Oxidative Stress and Antioxidant Status

Oxidative stress and antioxidant status were evaluated in isolated islets as previously reported (Yao et al., 2019a). After drug treatment, oxidative stress was determined by dihydroethidium (DHE) assay kit in live cells. The lipid peroxidation levels (malondialdehyde, MDA) and the activities of SOD and GPX were measured to evaluate the antioxidant status in medium supernatants after different drug treatments using detective kits (Nanjing Jiancheng, China).

### Determination of the Protein Expression

The protein levels of the protein expression of GPX4, HO-1, and Nrf2 were quantified by western blot analysis (Kou et al., 2019). Firstly, Min6 cells were digested and lysed before centrifugation (12,000 g, 4°C) for 10 min to collect protein. The BCA Protein Assay Kit was used to determine protein concentration. Total protein (20 μg per well) was electrophoresed on sodium dodecyl sulfate-polyacrylamide gels before transferring to polyvinylidene difluoride (PVDF) membranes. Nonspecific binding was blocked by 5% nonfat milk powder for 1.5 h. Then the samples were incubated with primary antibodies (GPX4, Abcam, ab125066; Nrf2, Abcam, ab137550; HO-1, Proteintech, 66743-1-Ig; β-actin, Cell Signaling, #3700S) overnight at 4 °C. Afterwards, the membranes was washed and then incubated with a secondary antibody (horseradish peroxidase-conjugated) before chemiluminescence detection using an Amersham prime ECL Plus detection system (Pittsburgh, PA).

### Intracellular Iron Measurement

The intracellular free iron concentration was measured by inductively-coupled plasma mass spectrometry (ICP-MS) (Agilent 7800). Min6 cells were firstly pretreated with erastin, then incubated with ferroptosis inhibitors or bilirubin. The treated cells were then lysed by probe sonification. The samples were centrifugated with a speed of 13000 g at 4°C for 30 min, and the supernatant was collected. Concentrated nitric acid was added into the supernatant sample, and the mixture was then heated for 2 hours at 80 °C for complete digestion. The iron concentration was then measured by ICP-MS under suitable conditions for routine multi-element analysis.

### UV-Vis Spectroscopic Analysis

The iron solution (Fe(NO_3_)_3_ or FeSO_4_) and bilirubin solutions were mixed appropriately to generate solutions of different molar fraction ratio between iron and bilirubin, and UV-Vis spectrometer (Cary 100, Agilent) was used to determine the stoichiometric properties of each complex at 25°C.

### IR Spectroscopic Analysis

The iron solution (Fe (NO_3_)_3_ or FeSO_4_) and bilirubin solutions were mixed appropriately to generate solutions of different molar fraction ratio between iron and bilirubin. Then the mixed solution was centrifuged at 7500 g. The sediments were measured with an IR spectrometer (Shimadzu, Japan) to collect the IR spectra. The scans were executed with a resolution of 2 cm^−1^ and from 4,000 cm^−1^ to 400 cm^−1^.

### *In Vivo* Islet Transplantation

BALB/c mouse are used as recipients and donors in the transplantation. A streptozotocin (STZ; Sigma)-induced diabetic BALB/c mouse model developed as reported in other studies was used in this study (Yao et al., 2019a; Yao et al., 2020a). Fifteen diabetic BALB/c mice were randomly divided into 5 groups (3 mice in each group). Then, 250 IEQ islets pretreated with or without bilirubin (20 μM) for 48 h were transplanted into the subrenal site of the diabetic mouse. Ferrin 1 (10 μM) and DFO (10 mM) pretreated islets were also transplanted for comparison. Following, the non-fasting glucose level and bodyweight was the mice were recorded daily. The investigators were blinded to the treatment and allocation during the experiments. All treatments and animal care procedures were performed in accordance with the Animal Care and Use Committee of Wenzhou Medical University.

We used the intraperitoneal glucose tolerance test (IPGTT) to evaluate the transplanted islet function in vivo. In brief, the transplanted mice were firstly fasted for 8 h, and then received intraperitoneal glucose injection (2 g/kg). After that, the blood glucose level was monitored in the following 2 h, and the area under curve (AUC) of blood glucose was calculated.

### Statistical Analysis

Statistical analysis was conducted with analysis of variance (ANOVA) method to compare data among groups, and *P* < 0.05 was considered significant.

## Results

### Ferroptosis Inducing Agents Caused Islet Death and Dysfunction

**Figure 1:**
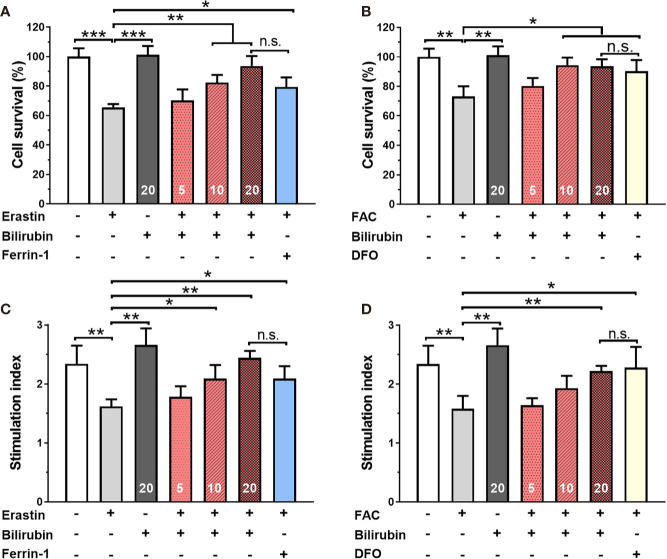
Bilirubin pretreatment improved the survival and insulin secretion in islets against erastin (50 μM) and over-load iron (10 mM). Islets were pretreated with or without bilirubin (5 μM, 10 μM, 20 μM, respectively) for 24 h and subsequently treated with erastin (50 μM) or FAC (10 mM) for another 24 h. Pretreatment with bilirubin dramatically increased islet viability after 50 μM erastin **(A)** or 10 mM FAC **(C)** treatment. The insulin secretion function of islets was retained after bilirubin pretreatment after 50 μM erastin **(B)** or 10 mM FAC treatment **(D)**. Data are presented as mean ± SD (n = 3). *P < 0.05, **P < 0.01, and ***P < 0.001.

Islets co-treated with 50 μM erastin or 10 mM ferric ammonium citrate (FAC) underwent significant cell death compared to those from the control group (**Figures 1A, B**) (50 μM erastin: 65.47%, *P*<0.001; 10 mM FAC: 73.25, *P*<0.01). As for insulin secretion function (**Figures 1C, D**), islets treated with erastin (50 μM) or FAC (10 mM) exhibited reduced insulin production ability upon glucose stimulation (SI: control 2.34±0.31 vs. 50 μM erastin 1.62±0.18 vs. 10 mM FAC 1.58±0.22, *P*<0.05). Collectively, these data confirmed that ferroptosis inducing agents could cause islet loss and dysfunction.

### Bilirubin Pretreatment Improved the Survival and Insulin Secretion Function in Islets Against Erastin or Overloaded Iron.

After 24 h 50 μM erastin treatment, islets pretreated with Ferrin-1 exhibited significantly high islet survival rate compared to only erastin-treated islets (*P*<0.05) (**Figure 1A**). Notably, islets pretreated with 5, 10, and 20 μM bilirubin also displayed significantly increased islet survival compared to only erastin-treated islets (N.S., *P*<0.01, and *P*<0.01, respectively), and the effect was even higher than that of the islets pretreated with Ferrin-1 at a concentration of 20 μM. We also evaluated the islet survival with or without bilirubin against 10 mM FAC (**Figure 1B**). DFO treatment significantly improved islet survival compared to only FAC-treated islets (*P*<0.05). In addition, islets pretreated with 5, 10, and 20 μM bilirubin also displayed increased survival compared to the islets treated with FAC alone (N.S., *P*<0.05, and *P*<0.05, respectively). These data indicated that bilirubin could protect the islet from erastin- or FAC-induced islet damage and loss, and the protective effect became more significant as the bilirubin concentration increased. We further evaluated whether bilirubin pretreatment could offset the ferroptosis inducing agents caused impaired insulin secretion function (**Figures 1C, D**). The results showed that 50 μM erastin significantly impaired islet function, as evidenced by compromised insulin secretion after high glucose stimulation (**Figure 1C**, *P*<0.01). While Ferrin-1 and DFO pretreatment could improve glucose-stimulated insulin secretion in the erastin (*P*<0.05) or FAC(*P*<0.05) impaired islets. As expected, the islets pretreated with bilirubin (5, 10, 20 μM) before erastin treatment exhibited considerable higher insulin secretion as compared to islets treated with erastin alone (N.S., *P*<0.05, and *P*<0.01, respectively). Bilirubin pretreatment maximumly maintained the insulin secretion capacity (SI 20 μM bilirubin: 2.22±0.09 vs. control 2.34±0.32, *P*<0.01) of islets in the presence of erastin (**Figure 1C**). Similar trends were also observed when FAC was introduced as a ferroptosis inducing agent (**Figure 1D**). These results indicated that bilirubin could prevent islets from ferroptosis induced islet loss and dysfunction in a concentration-dependent manner.

### Bilirubin Pretreatment Attenuated Ferroptosis By Enhancing ROS Scavenge and Antioxidative Enzyme Activity

**Figure 2:**
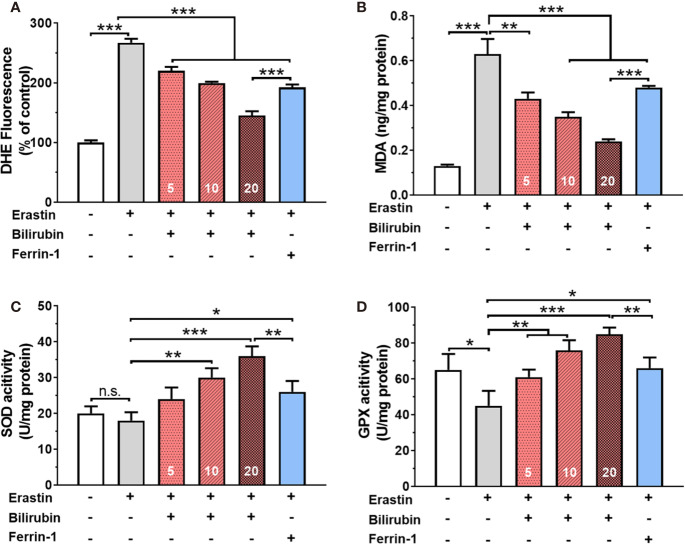
Oxidative stress and antioxidant status in islets after various drug treatments. The ROS levels **(A)**, MDA levels **(B)**, SOD activity **(C)**, and GPX activity **(D)** of the islets with bilirubin after 50 μM erastin treatment. Islets were pretreated with or without bilirubin for 24 h and then treated with 50 μM erastin for another 24 h. Afterward, the supernatant was collected for the assessment. Data are presented as mean ± SD (n = 3). *P < 0.05, **P < 0.01, and ***P < 0.001.

Twenty-four hours of 50 μM erastin treatment increased intracellular ROS levels up to 2.67 times in islets (**Figure 2A**). Pretreatment with bilirubin or ferroptosis inhibitor (Ferrin-1) could inhibit over 28% ROS production (*P*<0.001). Likewise, lipid peroxidation product MDA was also significantly increased while islets were exposed to 50 μM erastin. (control: 0.13±0.01, erastin, 0.63±0.07, *P*<0.001). Pretreated with bilirubin significantly decreased the MDA concentration in islets (**Figure 2B**) exposing to erastin compared to those with erastin treatment only (*P*<0.001). The activity of two antioxidative enzymes, SOD and GPX, was also evaluated (**Figures 2C, D**). In the presence of 50 μM erastin, the activity of SOD had no obvious changes while GPX activity was slightly compromised (control: 65.10±8.98, Erastin: 45.32±8.32, *P*<0.05). When bilirubin pretreatment was introduced, the activity of both antioxidative enzymes in islets was significantly enhanced at three tested concentrations (*P*<0.01). These data indicated that ferroptosis inducing agents could significantly increase ROS production and lipid peroxidation in islets, which could be attenuated by the pretreatment of bilirubin and Ferrin-1.

### Bilirubin Had Iron-Chelating Ability

**Figure 3:**
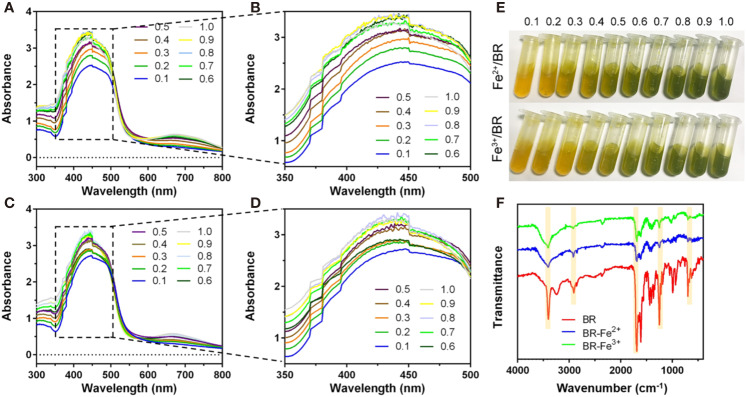
Iron chelation property of bilirubin. UV-Vis spectra for the chelation of Fe(II) **(A, B)** and Fe(III) **(C, D)** by bilirubin at the different molar ratios. **(E)** Images of formed bilirubin-iron complex at the different molar ratios. **(F)** IR spectra for Fe(II)-bilirubin and Fe(III)-bilirubin complexes.

The chelation of Fe(II) by bilirubin was initiated by mixing FeSO_4_ with bilirubin and letting the absorbance of the mixture reach a steady state. The UV-Vis spectra of the complex formation at different Fe(II)/bilirubin ratios were presented in **Figures 3A, B**. The absorbance of the complex increased as the Fe(II)/bilirubin ratio increased from 1:10 to 1:1 in the range from 350 nm to 500 nm (**Figures 3A, B**). The chelation of Fe(III) by bilirubin was initiated by mixing the respective Fe(NO_3_)_3_ with bilirubin. The chelation of Fe(III) by bilirubin exhibited a similar pattern (**Figures 3C, D**), and the absorbance increased along with the Fe(III)/bilirubin ratio. The images of iron/bilirubin complex (**Figure 3E**) showed that the color of the mixed solution went from light yellow to dark green, indicating the chelation between bilirubin and iron ion went stronger as the bilirubin/iron ratio increased. The IR spectral analysis of the chelation of Fe(II) and Fe(III) by bilirubin highlighted the shifting of the characteristic peaks of the carboxylate groups pointing the Fe(II)-oxygen bonding (**Figure 3F**). The ν(C=O) band of the carboxylate group in Fe(II)-bilirubin and Fe(III)-bilirubin shifted to a lower frequency (from 1636 cm^-1^ to 1538 cm^-1^ for Fe(II); from 1636 cm^-1^ to 1545 cm^-1^ for Fe(III), indicating the coordination of the carboxylate group in bilirubin to iron. The difference in the ν(C=O) band between the ligand and the ligand-metal complex was 98 cm^-1^ for Fe(II) and 91 cm^-1^ for Fe(III). For both Fe(II) and Fe(III), the coordinate bonding occurred through the carboxylate ion and the oxygen atom at 2-position in the bilirubin. As a result, the slight shift of the skeletal vibrations of the ligand in the complex could be observed. Decreased intensity of peaks at 1412.0 cm^-1^ and 1440.7 cm^-1^ for Fe(II)/bilirubin and Fe(II)/bilirubin complexes could also be seen. A similar pattern was observed for Fe(III). These results indicated that bilirubin had a strong iron-chelating ability.

**Figure 4:**
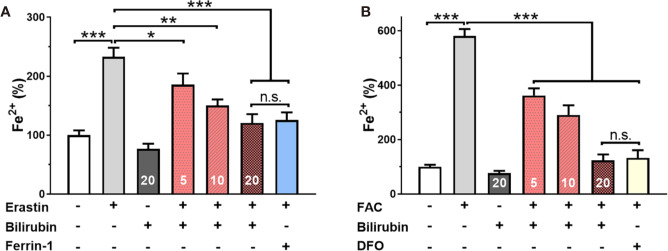
Bilirubin decreased intracellular iron level. Min6 cells were pretreated with or without bilirubin (5 μM, 10 μM, 20 μM, respectively) for 24 h and subsequently treated with erastin (50 μM) or FAC (10 mM) for another 24 h. ICP-MS was used to measure the intracellular iron concentration. Pretreatment with bilirubin dramatically decreased intracellular iron levels after 50 μM erastin **(A)** or 10 mM FAC **(B)** treatment. Data are presented as mean ± SD (n = 3). *P < 0.05, **P < 0.01, and ***P < 0.001.

To further confirm the iron-chelating ability of bilirubin in islets, the effects of ferroptosis inducer and inhibitor on the intracellular free iron were investigated by ICP-MS technique. As shown in **Figure 4A**, Min6 cells showed a significantly increased concentration of Fe^2+^ after 24-hour treatment of erastin. The pretreated bilirubin could suppress the intracellular iron compared to the erastin-treated Min6 cells, and this effect showed a concentration-dependent manner. It should be noted that bilirubin (20 μM) displayed a similar ability suppressing iron accumulation to Ferrin-1 (20 μM). When Min6 cells were only treated by bilirubin (20 μM), the concentration of intracellular iron could be further decreased compared to that in the untreated cells, confirming the iron-chelating ability of bilirubin. We also detected the intracellular iron accumulation of Min6 cells with or without bilirubin against 10 mM FAC (**Figure 4B**). DFO treatment could significantly moderate the increased intracellular iron level induced by FAC. In addition, Min6 cells pretreated with 5, 10, 20 μM bilirubin also decreased the intracellular iron accumulation compared to the islets treated with FAC alone (P<0.001). These data indicated that bilirubin could protect Min6 cells from intracellular iron accumulation, further confirming the strong iron-chelating ability of bilirubin.

### Bilirubin Inhibited Ferroptosis Through Upregulation of Nrf2/HO-1 Signaling Pathways

**Figure 5:**
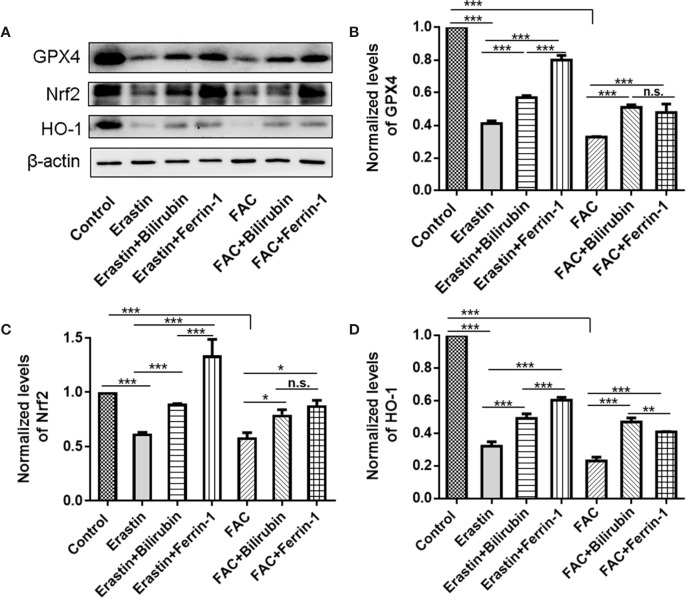
The protein expression **(A)** and semi-quantitative protein levels of GPX4 **(B)**, Nrf2 **(C)**, and HO-1**(D)** of the MIN6 pancreatic β cells with bilirubin in the presence of erastin (50 μM) or FAC (10 mM). Min6 cells were pretreated with or without bilirubin for 24 h and subsequently treated with erastin (50 μM) or FAC (10 mM) for an additional 24 h. Data are presented as mean ± SD (n = 3). *P < 0.05, **P < 0.01, and ***P < 0.001.

To address the role of Nrf2/HO-1 signaling pathway in the cytoprotective effects of bilirubin against ferroptosis, we examined the protein expression of Nrf2, HO-1, and GPX4 after various drug interventions (**Figure 5**). As indicated, the GPX4 protein expression was significantly decreased upon either erastin (*P*<0.001) or FAC treatment (*P*<0.001), indicating ferroptosis occurred. And the ferroptosis induced GPX4 down-regulation was significantly antagonized by Ferrin-1 and DFO treatment (*P*<0.001). This result was consistent with previously published reports that Ferrin-1 and DFO could inhibit ferroptosis in the pancreatic islets (Bruni et al., 2018b). The bilirubin treatment could also offset either erastin or FAC induced decreased GPX4 expression (*P*<0.001). Moreover, the addition of the ferroptosis inducing agents, either erastin or FAC, significantly reduced the Nrf2 and HO-1 expression (*P*<0.001), while this effect could be antagonized by the ferroptosis inhibitors (Ferrin-1 and DFO). Compared to the erastin group, cells treated with additional bilirubin showed significant increased expression of both Nrf2 (*P*<0.001) and HO-1 (*P*<0.001). Similarly, bilirubin pretreatment also upregulated the Nrf2 (*P*<0.05) and HO-1 (*P*<0.001) expression upon FAC treatment. These data indicated that protection role of bilirubin against ferroptosis involved the Nrf2/HO-1 signaling pathway.

### Bilirubin Protected the Transplanted Islet From Ferroptosis Induced Cell Death and Improved Graft Efficacy

**Figure 6:**
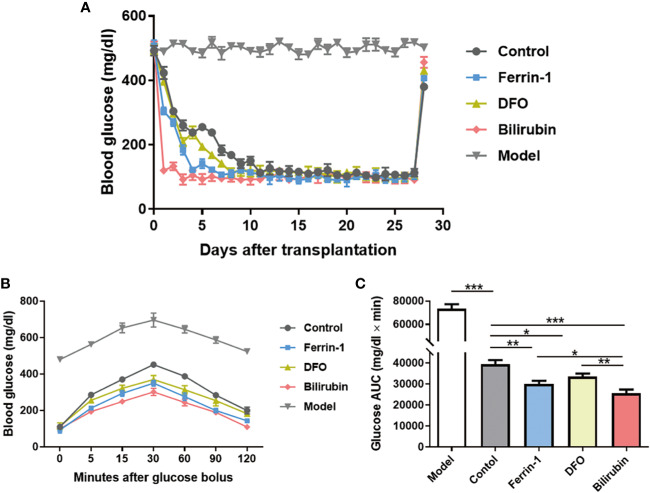
Efficacy of transplanted islets pretreated with bilirubin. The collected islets were pretreated with bilirubin (20 μM) for 48 h before transplanted under the kidney capsulate at a dose of 250 IEQs per diabetic recipient. Ferrin 1 and DFO pretreated islets were also transplanted for comparison. **(A)** Blood glucose changes after islet transplantation. **(B)** Blood glucose changes after glucose bolus on day 14. **(C)** The area under curves of glucose. Data are presented as mean ± SD, n = 3. *P < 0.05, **P < 0.01, and ***P < 0.001.

Islet transplantation outcomes of islets pretreated with or without bilirubin at a dose of 20 μM for 48 h were evaluated in an STZ-induced diabetic mouse model (250 IEQ per mice, n=3). Ferrin 1 and DFO pretreated islets were also transplanted for comparison. Model mice exhibited a high glucose concentration (>480 mg/dl) during the whole study. All recipients became euglycemia (<200 mg/dl) subsequent to transplant in all transplanted groups (**Figure 6A**). It is noticeable that diabetic mice received islets with bilirubin pretreatment required the least time (~1 day) to reach euglycemia among all grafted groups. Other groups that received ferroptosis inhibitor-pretreated islets also reached euglycemia faster than the non-treated islets group (Ferrin-1: ~3 days; DFO: ~5 days; control: ~7 days) (**Figure 6A**).

IPGTTs were also performed on mice 14 days post-transplantation. Diabetic mice received islet graft all exhibited a physiological response toward intraperitoneal bolus of glucose, as evidenced by the quick glucose removal from the circulation within 120 min (**Figure 6B**). However, the glucose area under the curve (AUC_120min_) among the islet graft groups exhibited a significant difference (AUC_120min_ control: 733410±3891 vs. bilirubin: 25647±1751, *P*<0.001, **Figure 6C**). Besides bilirubin, other ferroptosis inhibitors, Ferrin-1- and DFO-pretreated islets also exhibited decreased AUC_120min_ values (AUC_120min_ control: 733410±3891 vs. Ferrin-1: 30007±1608 vs. DFO: 33564±1440, *P* <0.01, *P*<0.05). More importantly, the protective effects of bilirubin on the transplanted islet function was more significant than those with Ferrin-1 (*P*<0.05) or DFO treatment (*P*<0.01). These experiments suggested that bilirubin and ferroptosis inhibitors (Ferrin-1 and DFO) pretreatment could enhance islet engraftment outcomes.

## Discussion

The main objective of this study was to determine whether bilirubin exerted protective effects on the transplanted islets by targeting ferroptosis. To our best knowledge, this is the very first study that confirms bilirubin could protect the isolated islets by antagonizing ferroptosis. It is of clinic interest to maximumly reduce the islet loss during the early post-transplantation period (Shapiro et al., 2017). During the islet collection, preservation, and transplantation, isolated islets always suffered extensive oxidative stress that significantly compromised the islet survival and function. It has been acknowledged that strategies that target islet redox signaling could potentially protect the islets from oxidative stress-induced damages (Li et al., 2019; Zhu et al., 2019). Ferroptosis, a form of programmed death, is characterized by the intensive oxidative stress and the involvement of iron. GSH is the most important intracellular antioxidant that proved to be able to reduce ROS accumulation (Kou et al., 2019; Kou et al., 2020). Erastin could inhibit cystine uptake by interfering xCT transporters, which reduced the intracellular GSH biosynthesis and induced ferroptosis (Park and Chung, 2019). More importantly, the direct addition of over-load iron, FAC, has also been identified as an effective ferroptosis inducing approach (Li et al., 2019). It is viable to increase islet survival and function by reducing oxidative stress (Yao et al., 2019b). Bruni et al. demonstrated that inhibiting ferroptosis by Ferrin-1 or iron chelator could protect isolated islets from lipid peroxidation induced impaired function (Bruni et al., 2018a; Bruni et al., 2018b). However, the role of ferroptosis in the islet transplantation still lacks enough investigation. Our previous study has shown that bilirubin could effectively decrease the ROS accumulation in islets in the presence of H_2_O_2_ (Yao et al., 2019a). In addition, bilirubin could also improve the whole antioxidant defence system by increasing the activity of antioxidant enzymes, like SOD and GPX, and decreasing the lipid peroxidation MDA (Yao et al., 2019a). Reduced oxidative stress and enhanced antioxidant systems have been associated with the protective effect of bilirubin in islet preservation and transplantation (Adin et al., 2017; Kim et al., 2017b; Yao et al., 2019a). But the correlation of bilirubin and ferroptosis has not yet been elucidated.

In our study, we pretreated islets with bilirubin, Ferrin-1, or DFO before exposing them against ferroptosis inducing agents, and measure the islet survival and function. We found that the addition of ferroptosis inducing agents significantly reduced the islet survival and insulin secretion ability, indicating that ferroptosis caused the islet loss and dysfunction. The transplantation of ferroptosis inhibitors along with islets resulted in improved graft efficacy, which indicated that ferroptosis occurred in the transplanted islets from the opposite view. Meantime, pretreatment of bilirubin reduced islet loss and maintained the function of insulin secretion; the effect was similar to that of Ferrin-1 or DFO. The cytoprotective role of bilirubin on islets survival and function has been well reported (Adin et al., 2017; Kim et al., 2017b; Yao et al., 2019a) . Kim et al. reported that pretreatment with bilirubin increases the islet morphology and insulin secretion (Kim et al., 2017b). Our previous study also demonstrated that protective effects of bilirubin on the islets involved the decreased ROS levels, and increased intracellular antioxidative enzyme expression (Yao et al., 2019a). The correlation between bilirubin and ferroptosis was first evaluated in this study. As compared to Ferrin-1 and DFO, bilirubin exerted similar cytoprotective effects against ferroptosis in terms of cell viability and insulin secretion.

To further investigate the role of bilirubin in ferroptosis impaired islets, we also evaluated the effect of bilirubin on the two main contributing factors, ROS, and iron, in ferroptosis. Upon the erastin, islets produced 2.67-fold ROS compared to that of the control group. As expected, the addition of Ferrin-1 significantly reduced the levels of ROS and peroxidative lipid, MDA, and elevated the activities of SOD and GPX. Consistent with our previous results (Adin et al., 2017; Kim et al., 2017b; Yao et al., 2019a), bilirubin could also help the islets to decrease the ROS levels in a dose-dependent manner. Additionally, similar to the Ferrin-1 group, pretreatment of bilirubin also decreased the lipid peroxidation and enhanced the intracellular antioxidative enzymes to combat ferroptosis. The chelating effects of bilirubin on the metal ion have been widely reported(Shukla et al., 2012; Maity et al., 2014), but have not yet been linked to the ferroptosis induced cell death. Iron is a key factor in ferroptosis, and the over-load iron has been regarded as a hallmark of ferroptosis (Dixon, 2017). Accordingly, the iron chelator, e.g., DFO, has been proved sufficient to protect cells from ferroptosis. We investigated the iron-chelating properties of bilirubin on the Fe(II) and Fe(III). Bilirubin could quickly chelate both Fe(II) and Fe(III), as evidenced by the IR and UV-visible spectroscopy results. ICP-MS further confirmed that bilirubin could decrease the intracellular iron levels in islets. This effect would reinforce the anti-ferroptosis effect of bilirubin.

Western blot analysis was conducted to further investigate the protective mechanisms of bilirubin in isolated islets. The GPX4 expression was decreased in Min6 cells exposure to erastin or FAC, which is consistent with previously reported study (Yang et al., 2014). GPX4 is a key regulator of ferroptosis, that could be reactivated by ferroptosis inhibitors. Bilirubin pretreatment could increase the GPX4 expression after ferroptosis caused cell injury, indicating that bilirubin could counteract the ferroptosis-induced effects. The Nrf2/HO-1 signaling has a complex regulatory mechanism in oxidative stress disease (Yao et al., 2020a; Yao et al., 2020b) and has been reported to be involved in the protective effects against ferroptosis (Jiang et al., 2020). Our results demonstrated that both Nrf2 and HO-1 expression were significantly decreased while the cells exposed to the ferroptosis inducers, which could be offset by the ferroptosis inhibitors. HO-1 plays a vital role in the bilirubin metabolism and could be upregulated by bilirubin pretreatment (Kawamura et al., 2005). Also, Qaisiya et al. has reported that bilirubin is an endogenous inducer of Nrf2 pathway and cytoprotective effects under hyperbilirubinemia are closely related to the activation of Nrf2 pathway (Qaisiya, 2017). We also observed that bilirubin pretreatment could increase Nrf2 and HO-1 expression in the ferroptosis-injured cells to exert protective effects. Whereas, Kwon et al. reported that HO-1 played an essential role in ferroptic cell death induced by iron dependent lipid peroxidation, which could upregulated by heme, resulted in an increased ROS production (Kwon et al., 2015). This effect attributed to the iron overload resulted from HO-1 mediated heme metabolism and was considered beneficial for cancer therapy (Chiang et al., 2018). In this study, islets were transplanted into the subrenal site of the diabetic mouse, and heme could not distribute to the transplanted site by circulation. Therefore, the increased HO-1 after pretreatment with bilirubin could not induce iron overload and excessive ROS production.

Finally, we verified the protection of bilirubin on the transplanted islets in vivo. The blood glucose of bilirubin-treated group dropped to the normal level more quickly than those with Ferrin-1 or DFO pretreatment. Glucose tolerance results also showed that bilirubin-pretreated islets achieved best glucose removal ability, indicating that bilirubin-pretreated islets kept the islet function post-transplantation. It is surprising that bilirubin-pretreated islets achieved best islet graft outcome among the four treatment groups, even better than Ferrin-1 and DFO groups. It could be explained by the following reasons: 1) ferroptosis only partially contributed to the islet death and dysfunction in vivo, but many other factors, including lack of nutrition, hypoxia, inflammation, and host rejection, collectively cased the islet graft failure; 2) bilirubin could protect the islets not only by ferroptosis inhibition, but also through multiple effects, e.g., anti-oxidative stress property, ant-inflammation, and also immune modulation; 3) bilirubin sensitize the produced insulin and enhance the hypoglycemic activity.

**Figure 7:**
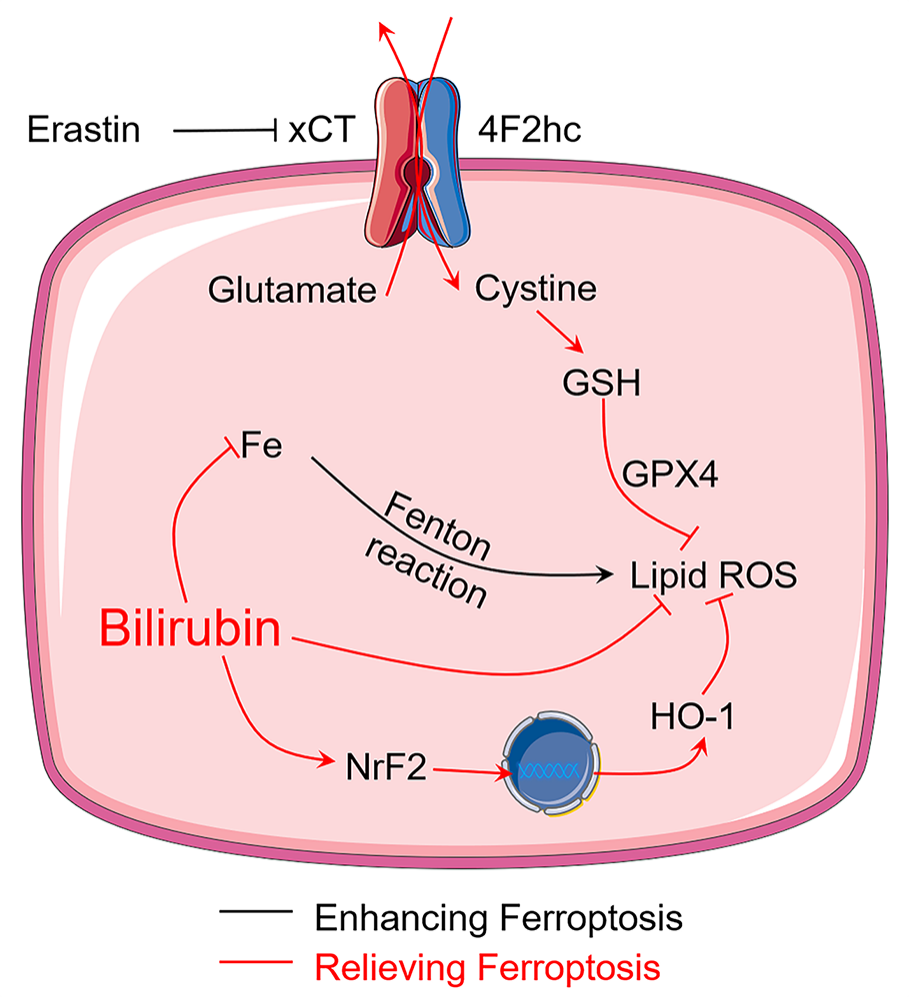
Bilirubin protects transplanted islets by inhibiting ferroptosis through multiple mechanisms, including ROS scavenging ability, iron-chelating property, and upregulation of Nrf2/HO-1 signaling pathway.

Although previous studies have demonstrated that bilirubin is beneficial for diabetes and islet transplantation (Adin et al., 2017; Kim et al., 2017b; Yao et al., 2019a), the question that whether bilirubin exerted its protective effects by inhibiting ferroptosis has never been answered. This study first reported that bilirubin could exert protective effects on isolated islets by inhibiting ferroptosis, which significantly increased the islet survival and function. As summarized in **Figure 7**, bilirubin could inhibit ferroptosis by 1) attenuating the oxidative stress through its ability of scavenging ROS; 2) reducing the intracellular iron level through iron-chelating property; and 3) upregulating the antioxidant Nrf2/HO-1 signaling pathway. Islet transplantation has been explored to be a treatment for type I diabetes in clinical researches. Islet loss during the preservation and the early post-transplantation period still hampered further development of islet transplantation. Bilirubin has shown great promise in the preservation and transplantation process by inhibiting ferroptosis, suppressing ROS level, decreasing inflammation, and immune modulation. In the future, more mechanism studies of bilirubin's cytoprotective effects on the transplanted islets should be conducted to provide more evidence for the application of bilirubin in the islet transplantation to advance the clinical application. Also, our ongoing research also indicated that with more formulation strategies that address the insolubility issue could further improve the transplantation outcomes of bilirubin-pretreated islets (Yao et al., 2020a).

## Conclusion

In summary, our data show that treating islets with bilirubin could improve the islet survival and function against ferroptosis through attenuated oxidative stress, iron chelation, and upregulation of Nrf2/HO-1 signaling pathways. The data also showed that bilirubin treatment improved islet graft efficiency, reducing the times to normalize blood glucose levels to as less as 24 h. The in vivo beneficial results could be explained by the effects observed in vitro. Our results provided mechanistic information and supported the use of bilirubin in transplanted islet to maximumly maintain its viability and insulin secretion function.

## Data Availability Statement

The datasets generated for this study are available on request to the corresponding authors.

## Ethics Statement

The animal study was reviewed and approved by Wenzhou Medical University.

## Author Contributions

LK and QY conceived the conception and design. QY did the most experiments and data analysis. RS did the western blot experiments and analysis. SB participated the discussion of this project. QY drafted the manuscript, and LK reviewed it. RC supervised the whole project. All authors contributed to the article and approved the submitted version.

## Funding

This research was supported by the National Natural Science Foundation of China (Grant 81903551, and 81803443), Key Research and Development Program of Zhejiang Province (Grant No. 2018C03013), Zhejiang Province Natural Science Foundation (Grant No. LQ19H300001), Wenzhou Municipal Science and Technology Bureau (Grant No. Y20190177, ZY2019007, Y20180180, Y20180208), and the Special Project for Significant New Drug Research and Development in the Major National Science and Technology Projects of China (2020ZX09201002).

## Conflict of Interest

The authors declare that the research was conducted in the absence of any commercial or financial relationships that could be construed as a potential conflict of interest.
